# Evaluation of the Effects of Quercetin on Damaged Salivary Secretion

**DOI:** 10.1371/journal.pone.0116008

**Published:** 2015-01-28

**Authors:** Ayako Takahashi, Hiroko Inoue, Kenji Mishima, Fumio Ide, Ryoko Nakayama, Ayaka Hasaka, Koufuchi Ryo, Yumi Ito, Takashi Sakurai, Yoshinori Hasegawa, Ichiro Saito

**Affiliations:** 1 Department of Pathology, Tsurumi University School of Dental Medicine, Yokohama, Japan; 2 Department of Pharmaceutical Sciences, Nihon Pharmaceutical University, Saitama, Japan; 3 Division of Pathology, Department of Oral Diagnostic Sciences, School of Dentistry, Showa University, Tokyo, Japan; 4 Department of Radiopraxis Science, Graduate School of Dentistry, Kanagawa Dental University, Yokosuka, Japan; 5 Department of Human Genome Research, Kazusa DNA Research Institute, Chiba, Japan; National Institutes of Health, UNITED STATES

## Abstract

With the aim of discovering an effective method to treat dry mouth, we analyzed the effects of quercetin on salivary secretion and its mechanism of action. We created a mouse model with impaired salivary secretion by exposure to radiation and found that impaired secretion is suppressed by quercetin intake. Moreover, secretion levels were enhanced in quercetin-fed normal mice. To elucidate the mechanisms of these effects on salivary secretion, we conducted an analysis using mouse submandibular gland tissues, a human salivary gland epithelial cell line (HSY), and mouse aortic endothelial cells (MAECs). The results showed that quercetin augments aquaporin 5 (AQP5) expression and calcium uptake, and suppresses oxidative stress and inflammatory responses induced by radiation exposure, suggesting that quercetin intake may be an effective method to treat impaired salivary secretion.

## Introduction

Dry mouth, which is caused by decreased salivary secretion levels, exhibits marked dryness and is known to be a risk factor for infections and aspiration pneumonia [1].

Several causes of impaired salivary secretion have been reported, including radiation therapy to the head and neck area, as well as Sjögren’s syndrome, an autoimmune disease. Moreover, chronic inflammation in localized gland tissue [2], involvement of oxidative stress [3], and microvascular disorders of the salivary gland [4] have also been reported as mechanisms of impairment. Quercetin is a polyphenol that has been shown to possess antioxidant properties [5] and anti-inflammatory [6] and vasodilatory effects [7, 8] and has been shown to promote angiogenesis [9]; therefore, effects on various diseases, such as ischemic disorders and hypertension, can be anticipated. Previous studies have reported that catechin, a polyphenol, is effective against radiation-induced salivary gland impairment [10] and that resveratrol, another polyphenol, suppresses reductions in salivary secretion [11]. However, the detailed effects of quercetin on salivary secretion have not been clarified. Therefore, in the present study, we used a mouse model with impaired salivary secretion to investigate the effects of quercetin on salivary secretion and analyzed its mechanism of action via in vitro experiments.

## Methods

### Animals

Six-week-old male C57BL6J mice (body weight 20–25 g; Clea Japan Inc., Tokyo, Japan) were used. Mice were housed in polycarbonate cages in a specific-pathogen-free (SPF) mouse colony and were given food and water. All animal experimental procedures were approved by the animal welfare committee of Tsurumi University (Kanagawa, Japan). The mice were anesthetized with a mixture of 10mg/kg xylazine and 100mg/kg ketamine intraperitoneally and then sacrificed humanely.

### Treatment groups and administration of quercetin

To evenly group mice by saliva amount, salivary secretion levels were measured prior to the start of the experiment, and mice were subsequently divided into 4 groups as follows: control group (fed a normal diet), irradiation group (15 Gy radiation), quercetin-fed group (1.25 g/kg/day or 0.25 g/kg/day), and quercetin + irradiation group (1.25 g/kg/day or 0.25 g/kg/day, and 15 Gy radiation). The quercetin dose was based on the outcome of a previous study [12, 13]. Mice were preventatively fed a dose of 1.25 g/kg/day starting 2 weeks prior to irradiation and were subsequently given 0.25 g/kg/day *ad libitum* after irradiation to suppress the long-term effects of quercetin administration. Quercetin was purchased from Sigma-Aldrich (catalog #Q4951; St. Louis, MO, USA).

### Irradiation

Each mouse was anesthetized by an intraperitoneal injection of a mixture of xylazine (28 mg/kg) and ketamine (63 mg/kg). Single acute exposure to 6 MV X-rays using a linear accelerator radiation therapy system (HL-1500; Hitachi Medical Corporation, Tokyo, Japan) was carried out at a dose rate of 2 Gy/min at a distance of 1000 mm [14]. The effective radiation dose to the submandibular gland (SMG) was set using the percentage depth dose and was greater than 95% of the maximum dose delivered.

### Measurement of salivary secretion

Salivary secretion was measured by collecting samples once a week, starting at quercetin administration, for 10 weeks. Mice were weighed and anesthetized by an intraperitoneal injection of a mixture of xylazine (24 mg/kg) and ketamine (36 mg/kg). After 10 min, pilocarpine (0.1 mg/kg) was injected intraperitoneally to stimulate salivation. The saliva secreted into the oral cavity during each 1-min period following injection of either of the above stimulants was carefully collected using capillary tubes (Ringcaps; Hirschmann Laborgerate GmbH & Co. KG, Eberstadt, Germany). The amount of total saliva collected in 15 min was divided by the weight of the mouse [14].

### Cell culture

Cultures were maintained in 100-mm culture dishes in a humidified atmosphere containing 95% air/5% CO_2_ at 37°C. Human salivary gland epithelial cell line (HSY) derived from an adenocarcinoma of the parotid gland were used (provided by Dr. M. Sato, Tokushima University) [15]. HSY cells were cultured in growth medium composed of DMEM (Sigma-Aldrich), 10% fetal bovine serum (FBS) (Sigma-Aldrich) and 1% penicillin-streptomycin (Wako, Osaka, Japan). A cell line derived from the aorta of a p53-deficient mouse (MAECs) was prepared by Nishiyama et al. and cultured in M199 medium (Sigma-Aldrich) with 5 ng/mL recombinant vascular endothelial growth factor (Sigma-Aldrich), 10 mM HEPES (Invitrogen, Tokyo, Japan), 1 U/mL heparin sodium (Shimizu, Shizuoka, Japan), 1% penicillin-streptomycin and 5% FBS [16]. The cell lines were allowed to grow to 80% confluence and were dissociated by a trypsin-EDTA solution followed by reseeding into a 100-mm culture dish and cultured for 24 h before use.

### Measurement of intracellular Ca^2+^ concentration

HSY cells were loaded with fura-2 by incubation for 20 min at 37°C with 3 μM fura-2-acetoxymethyl ester (Dojindo, Kumamoto, Japan) suspended in BSS-BSA, rinsed twice, resuspended in 5 mL of BSS-BSA, and stored at 4°C. Fura-2-loaded HSY cells were transferred to a 96-well black plate (Biomedical Science, Tokyo, Japan) and alternately illuminated by excitation at 340 and 380 nm. A microplate reader (Wallac 1420 ARVO SX Multilabel Counter, Perkin Elmer Co., Ltd., Massachusetts, USA) was used for measurements. Prior to measurements, cells were incubated at 37°C for 5 min and pre-treated with quercetin (1 μM, 10 μM, 100 μM) for 2 minutes. Subsequently, carbachol (cch) (30 μM or 100 μM) was added directly to the cell suspension during fluorescence measurements. The results are expressed as the fluorescence ratio (F340/F380). Fold changes of intracellular Ca^2+^ concentration were calculated using the values before and after cch injection.

### Radiation and quercetin treatment

MAECs were seeded at 0.3 × 10^6^ cells/well on 6-well plates and were incubated overnight at 37°C. On the following day, when the cells were at 80% confluence, the cell culture media was changed to serum-free media, and quercetin (50 μM, 100 μM) was added. As a control, 0.1% DMSO was added in place of quercetin. Twenty minutes after quercetin or 0.1% DMSO addition, the cells were irradiated with 30 Gy (MBR-1520R-3; Hitachi Power Solutions Co., Ltd., Ibaraki, Japan) of radiation and incubated at 37°C. The cells were collected 24 hours after irradiation.

### RNA extraction and gene expression analysis of various related genes by real-time RT-PCR

RNA was extracted from harvested mouse submandibular glands (SMGs) using the TRIzol reagent (Invitrogen). RNA was similarly extracted from irradiated or quercetin-treated MAECs. cDNA was synthesized from 2.5 μg of RNA using a SuperScript VILO cDNA Synthesis Kit (Invitrogen). Real-time PCR analysis was performed using the primers shown in Table 1. Reactions were carried out using the Step One Plus real-time PCR system (Applied Biosystems, Tokyo, Japan) and SYBR Premix Ex Taq II (Tli RNaseH Plus) (TAKARA BIO INC., Shiga, Japan) using the following procedure: 30 s at 95°C followed by 40 rounds of 5 s at 95°C and 34 s at 60°C. To verify that primer pairs produced only a single product, the temperature was gradually increased from 60°C to 95°C after the PCR for melting curve analysis. The eNOS mRNA expression was analyzed by RT-PCR using the primers shown in Table 1 using the following procedure: denaturation at 94°C for 30 s, annealing at 60°C for 30 s, and extension at 72°C for 1 min, for 30 cycles. The results for each mRNA level were normalized against β-actin.

**Table 1:** Primers used for Real-time RT-PCR (1) and RT-PCR (2).

Primer name	Primer sequence (5′-3′)		
TNF-α	sense	TGAAGGGGAATGGGTGTTCAT	(1)
	antisense	TTGGACCCTGAGCCATAATC	
IL-10	sense	TGCACTACCAAAGCCACAAG	(1)
	antisense	TAAGAGCAGGCAGCATAGCA	
AQP5	sense	TGGAGCAGGCATCCTGTACT	(1)
	antisense	CGTGGAGGAGAAGATGCAGA	
M3R	sense	GGTAGGTGAGTGGCCTGGTA	(1)
	antisense	GACACCTCCAGTGACCCTCT	
eNOS	sense	GATGAGTATGATGTGGTGTCCC	(2)
	antisense	GCCTAGGGGAGCTGTTGTA	
β-actin	sense	TGTTACCAACTGGGACGACA	(1)(2)
	antisense	CTGGGTCATCTTTTCACGGT	

### MDA detection in mouse SMGs

Mouse SMGs were placed into 1.5-mL serum tubes containing phosphate buffer (pH 7.0, with EDTA) at a density of 9-fold per tissue weight, and butylated hydroxytoluene (BHT) was added at a concentration of 0.1% to inhibit peroxidation. The tissues were homogenized using a Bio-Masher II^@^ (Nippi, Tokyo, JAPAN), centrifuged for 5 minutes at 10,000 × *g*, and the supernatants were removed and used.

MDA levels in mouse SMGs were measured using a NWLSS Malondialdehyde Assay kit (Northwest Life Science Specialties, Canada) following the manufacturer’s instructions.

Hemoglobin was removed following the procedure shown below. An equal volume of 1-butanol was added to the sample and allowed to react with indicator. The mixture was vortexed and centrifuged, and the butanol layer was collected. An equal volume of 1 M NaOH was added to the butanol layer, and the mixture was vortexed and centrifuged. The aqueous layer was collected as the measurement sample. The absorption spectrum (400–700 nm) of the sample was measured every 1 nm using a DU800 Spectrophotometer (Beckman Instruments Coulter Corporation, Tokyo, Japan).

Analysis was conducted using the 3^rd^ derivative analysis method described by Botsoglou et al. [17]. The protein content of the tissue homogenates was quantified using a BCA Protein Assay kit (Thermo Fisher Scientific K.K., USA), and the MDA levels in mouse SMGs were expressed in pmol MDA/mg protein.

### Gene cascade analysis

Because impaired salivary secretion can occur as a result of impaired blood flow [4], a comprehensive gene cascade analysis on quercetin-treated (50 μM) MAECs was conducted. The RNA-seq of the extracted RNA was performed using an Illumina HiSeq (Illumina K.K., Tokyo, Japan). Subsequently, gene expression cascade analysis was conducted on the fragments obtained per kilobase of exon per million mapped fragments (FPKM) values, and genes related to quercetin treatment were analyzed. Analysis was conducted in 2 steps: with transcription factor binding site analysis (TFBS analysis) and with key node analysis. Following the method described by Dillies et al., FPKM values were normalized against the trimmed mean of M-values (TMM) method [18] and were extracted into 2 groups: genes that showed changes (Yes-set) and those that did not show changes (No-set). TFBSs included in both sets were compared, and binding sites that were included significantly in the Yes-set were searched in the BIOBASE TRANSFAC Professional database (Biobase GmbH, Wolfenbüttel, Germany). TFBS analysis was performed to predict the binding factors and each gene. Furthermore, to search for factors that influence changes in gene expression (key node), key node analysis was performed by extracting the Yes-set from the obtained related genes and searching for related factors upstream of the factors in a stepwise manner.

### Statistics

Each experiment was repeated three times. Data were analyzed by one-way ANOVA, and then differences among means in eNOS expression analysis and intracellular Ca^2+^ concentration measurement or the others were analyzed using Dunnett’s or Tukey-Kramer multiple comparison tests, respectively. A value of <0.05 was regarded as being statistically significant. All values are expressed as the mean ± SD.

## Results

### Investigation of saliva amount and the effects of quercetin in a mouse model with impaired salivary secretion

**Figure 1:**
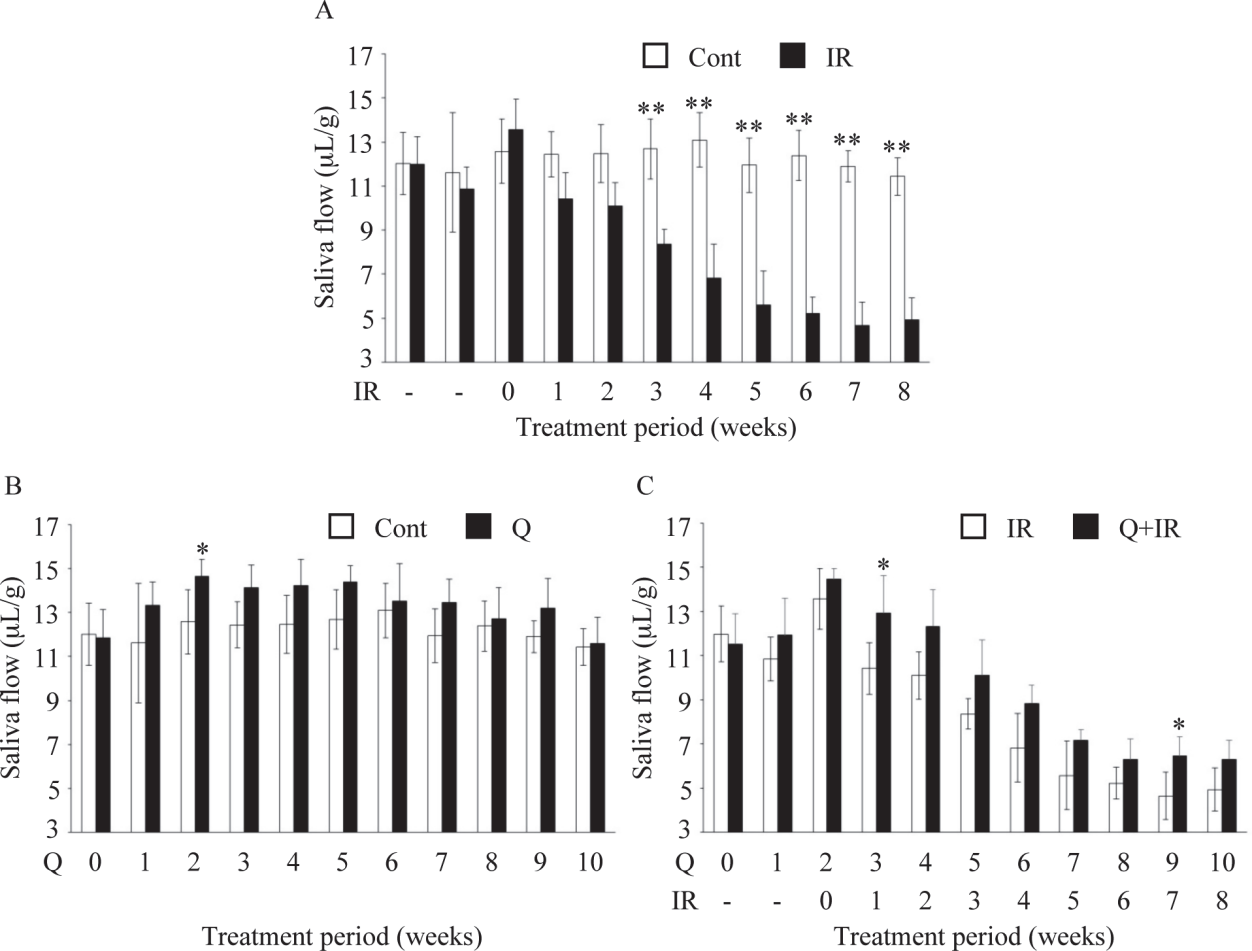
Effects of quercetin on salivary secretion. Quercetin was given at a dose of 1.25 g/kg/day for 2 weeks from the start of the experiment and subsequently at 0.25 g/kg/day for 8 weeks. Radiation exposure was conducted 2 weeks after the start of the experiment. Salivary secretion levels were measured for 15 min after pilocarpine stimulation, and the total saliva amount was corrected for body weight. Data are shown in terms of salivary secretion levels per g body weight. (A) Comparison between control group (fed a normal diet, Cont) and irradiated group (15 Gy radiation, IR). (B) Comparison between quercetin-fed group (1.25 g/kg/day or 0.25 g/kg/day, Q) and control group. (C) Comparison between quercetin + irradiated group (1.25 g/kg/day or 0.25 g/kg/day and 15 Gy radiation, Q+IR) and exposure group. Data are shown as the mean ± standard deviation (n = 5). Significant differences are expressed as *P <0.05, **P <0.01.

In the irradiated group, a decrease in salivary secretion levels was observed in a time-dependent manner after irradiation, and the amount was at its lowest at 7 weeks after irradiation (4.65 ± 1.07 μL/g). Compared to the control group, the salivary secretion levels were significantly lower from 3 week after starting until completion (Fig. 1A), and these results were in agreement with previous reports that studied the radiation-induced impaired salivary secretion in mice [14].

Salivary secretion was increased in quercetin-fed irradiated mice, as shown by the significant decrease in levels 3 and 9 weeks after intake (Fig. 1C). In addition, significant increase in salivary secretion levels was observed in non-irradiated normal mice at 2 weeks after quercetin intake (Fig. 1B).

### Inflammatory cytokine gene expression analysis in SMGs

**Figure 2:**
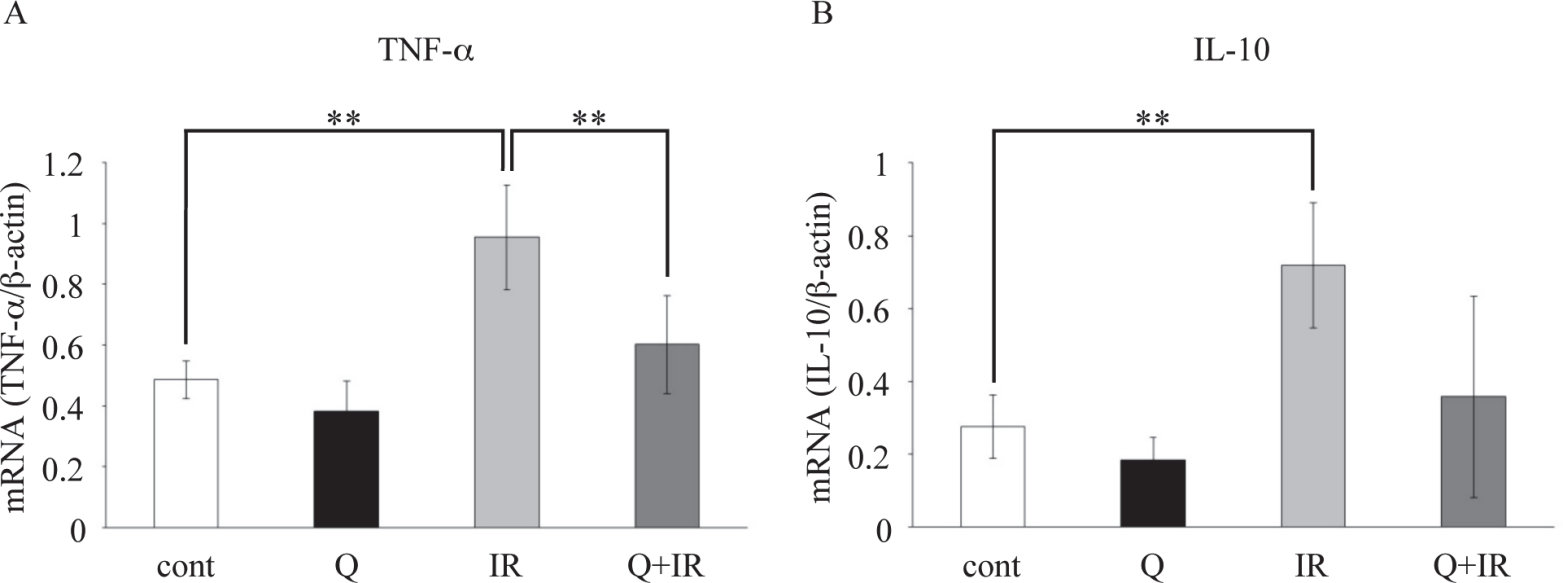
Effects of quercetin on proinflammatory cytokine expression in SMGs. SMG tissues from each group were harvested 1 week after the completion of the salivary secretion measurement, and RNA samples extracted from these tissues were used for real-time RT-PCR quantification. (A) TNF-α expression levels are shown. (B) IL-10 expression levels are shown. β-actin was used as an internal control. Data are shown as the mean ± standard deviation (n = 5). Significant differences are expressed as **P <0.01. Cont, fed a normal diet; Q, fed quercetin (1.25 g/kg/day or 0.25 g/kg/day); IR, 15 Gy radiation; Q+IR, fed quercetin plus 15 Gy radiation.

To investigate quercetin’s anti-inflammatory effects in gland tissues, we examined the gene expression of inflammatory cytokines in mouse SMG tissues. Our findings demonstrated that while the irradiated group had significantly elevated TNF-α and IL-10 expression, the quercetin-fed group showed a clear decrease in TNF-α expression when compared to the groups that were not given quercetin, and IL-10 similarly showed a decreasing trend in the quercetin-fed group (Fig. 2A & B). These results indicate that quercetin suppresses the expression of inflammatory cytokines induced by radiation.

### Gene expression analysis of molecules related to salivary secretion

**Figure 3:**
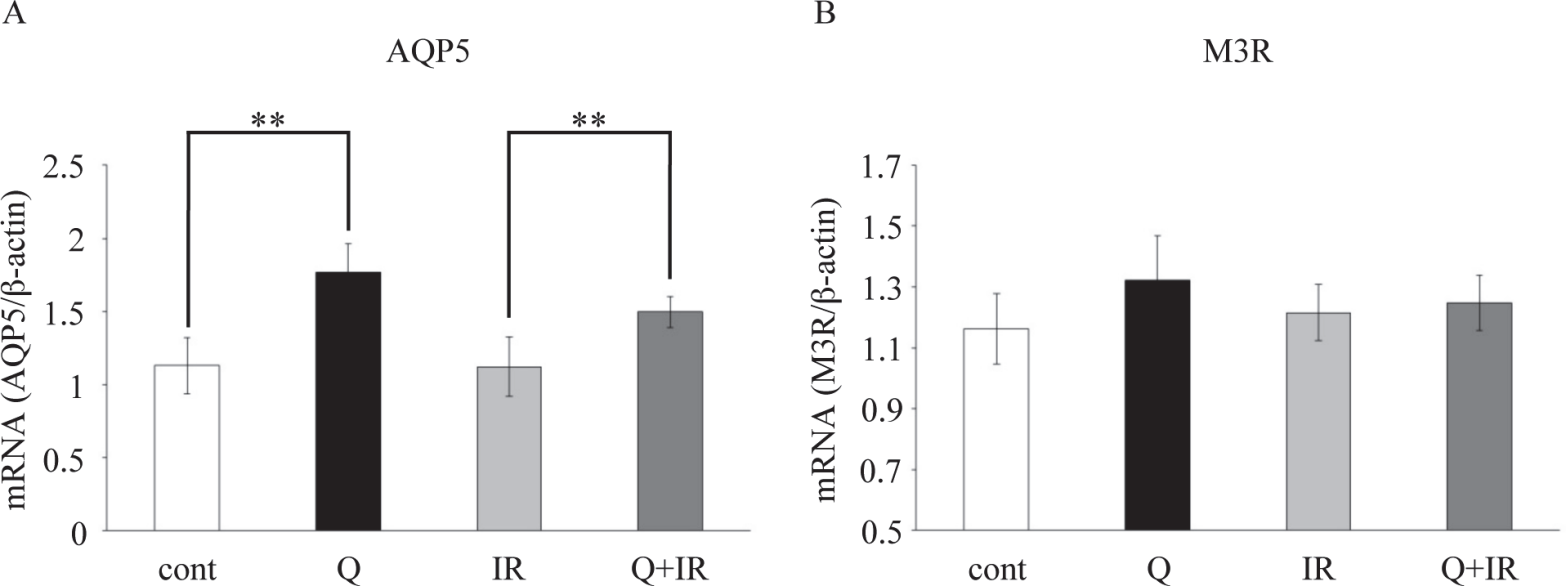
Effects of quercetin on the expression of salivary secretion-related molecules in SMGs. SMG tissues from each group were harvested 1 week after the completion of the salivary secretion measurement, and RNA samples extracted from these tissues were used for real-time RT-PCR quantification. (A) AQP5 expression levels are shown. (B) M3R expression levels are shown. β-actin was used as an internal control. Data are shown as the mean ± standard deviation (n = 5). Significant differences are expressed as **P <0.01. Cont, fed a normal diet; Q, fed quercetin (1.25 g/kg/day or 0.25 g/kg/day); IR, 15 Gy radiation; Q+IR, fed quercetin plus 15 Gy radiation.

In the present study, salivary secretion levels increased in quercetin-fed normal mice. For this reason, we examined Aquaporin 5 (AQP5) and Muscarinic type 3 receptor (M3R) gene expression to determine whether quercetin acts on molecules related to salivary secretion. In the SMGs of quercetin-fed normal mice, AQP5 expression significantly increased compared to the AQP5 expression in control mice. Similarly, in irradiated mice, quercetin intake clearly upregulated AQP5 compared to that in animals that did not receive quercetin (Fig. 3A). In addition, a trend in increasing M3R expression was observed in quercetin-fed normal mice (Fig. 3B), suggesting that quercetin may augment the expression of molecules related to water secretion from the SMG.

### Intracellular Ca^2+^ concentration

**Figure 4:**
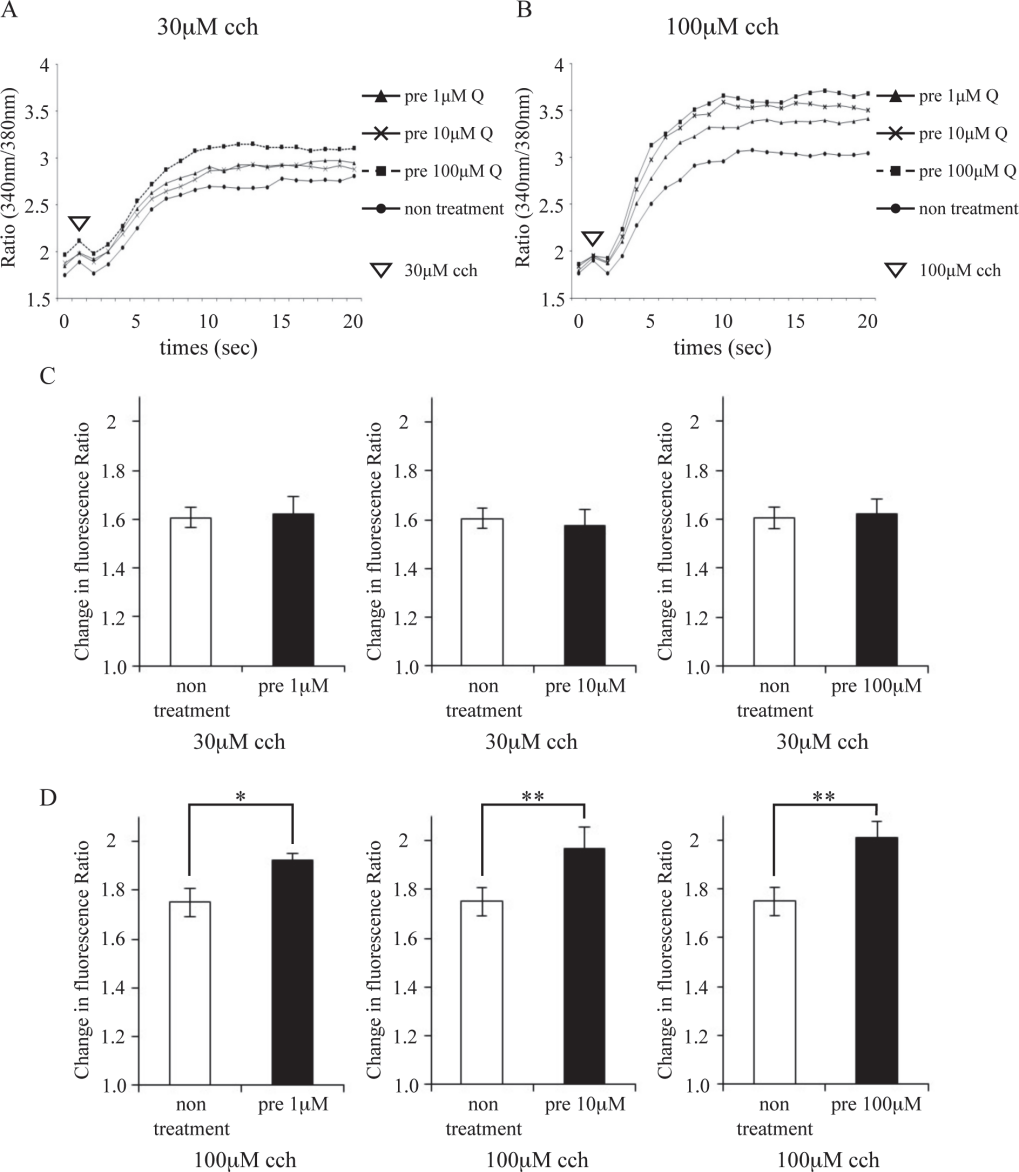
Effects of quercetin on calcium uptake into HSY cells. Carbachol (cch)-induced intracellular calcium concentrations were measured in HSY cells that were pretreated with quercetin (Q, 1 μM, 10 μM, 100 μM) for 2 minutes or left untreated. (A, B) Time points at which cch was added onto fura-2-loaded cells are indicated by arrows. (A) Changes in intracellular calcium upon 30 μM cch stimulation are shown. (B) Changes in intracellular calcium upon 100 μM cch stimulation are shown. (C, D) Changes in the ratios of baseline values prior to cch stimulation and top peak values after stimulation are shown. (C) Upon 30 μM cch stimulation. (D) Upon 100 μM cch stimulation. Data are shown as the mean ± standard deviation (n = 3). Significant differences are expressed as *P <0.05, **P <0.01.

Because it has been suggested *in vivo* that quercetin induces salivary secretion, we used HSY cells to confirm the changes in SMG intracellular Ca^2+^ concentration ([Ca^2+^]_i_) by quercetin treatment in vitro. Treatment with 100 μM cch, a muscarinic receptor agonist that is known to stimulate water secretion, significantly increased Ca^2+^ concentration in a dose-dependent manner in quercetin-treated cells compared to that in cells that were not treated with quercetin (Fig. 4D). These results suggest that quercetin promotes salivary secretion by enhancing intracellular calcium uptake in the SMG.

### Detection of eNOS expression levels

**Figure 5:**
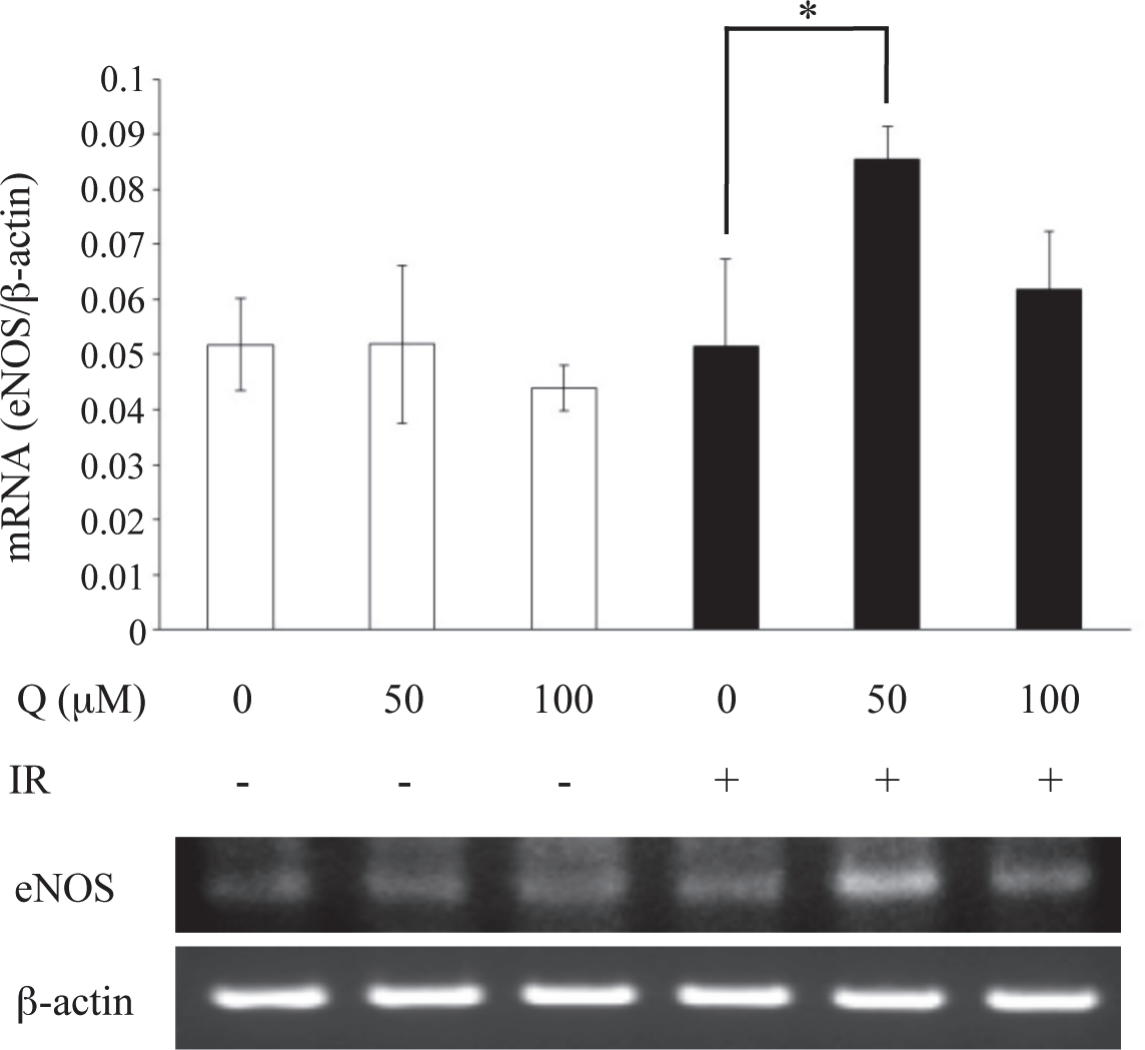
Effects of quercetin on eNOS expression in MAECs. Samples of RNA extracted from radiation-exposed (IR, 30 Gy) and quercetin-treated (Q, 50 μM, 100 μM) MAECs were used for RT-PCR, and band intensities were quantified using Quantity One 1-D software (Bio-Rad, Hercules, CA). β-actin was used as an internal control. Data are shown as the mean ± standard deviation (n = 5). Significant differences are expressed as *P <0.05.

Traditionally, quercetin is known to possess vasodilatory effects [7]. Because it is possible that peripheral circulation improved through this route, thereby promoting salivary secretion, we investigated the effects of quercetin on eNOS expression using MAEC, a mouse vascular endothelial cell line. The results showed that quercetin treatment increased eNOS expression in irradiated cells, but cells that were not irradiated did not exhibit changes in eNOS (Fig. 5). In addition, cells that were not treated with quercetin had similar eNOS expression, irrespective of irradiation (Fig. 5). These findings indicate that quercetin inhibits eNOS downregulation induced by radiation.

### MDA detection in mouse SMGs

**Figure 6:**
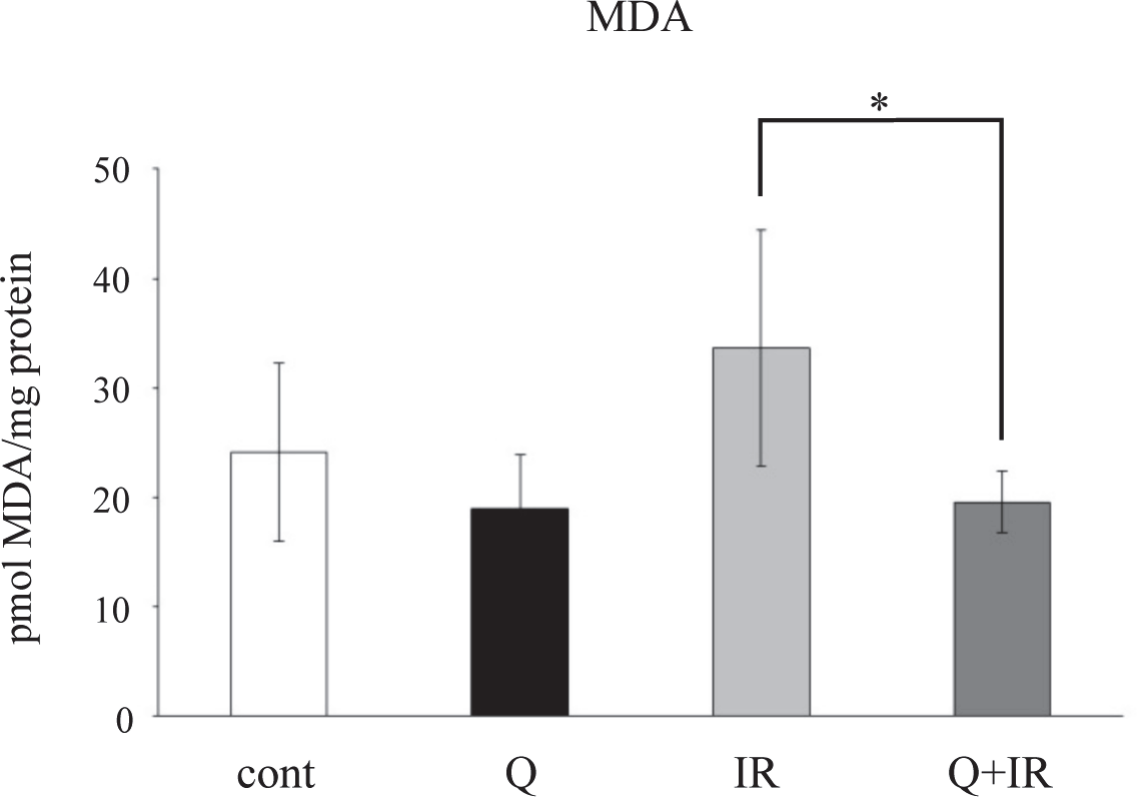
Effects of quercetin on MDA. SMG tissues from each group were harvested one week after completion of the salivary secretion measurements and were used for MDA measurements. Total protein in SMG tissues was used in the MDA detection assessment. Data are shown as the mean ± standard deviation (n = 5). Significant differences are expressed as *P <0.05. Cont, fed a normal diet; Q, fed quercetin (1.25 g/kg/day or 0.25 g/kg/day); IR, 15 Gy radiation; Q+IR, fed quercetin plus 15 Gy radiation.

Because impaired salivary secretion is presumed to occur through oxidative stress caused by radiation exposure, we investigated whether quercetin suppresses oxidative stress. Using mouse SMG tissue extracts, we assessed the amount of the oxidative stress marker MDA. The results showed that while MDA exhibited an increasing trend upon irradiation in the group of mice that were not given quercetin, the quercetin-fed group had significantly lower MDA (Fig. 6). These observations indicate that quercetin suppresses MDA generated by radiation exposure.

### Gene expression cascade analysis

**Figure 7:**
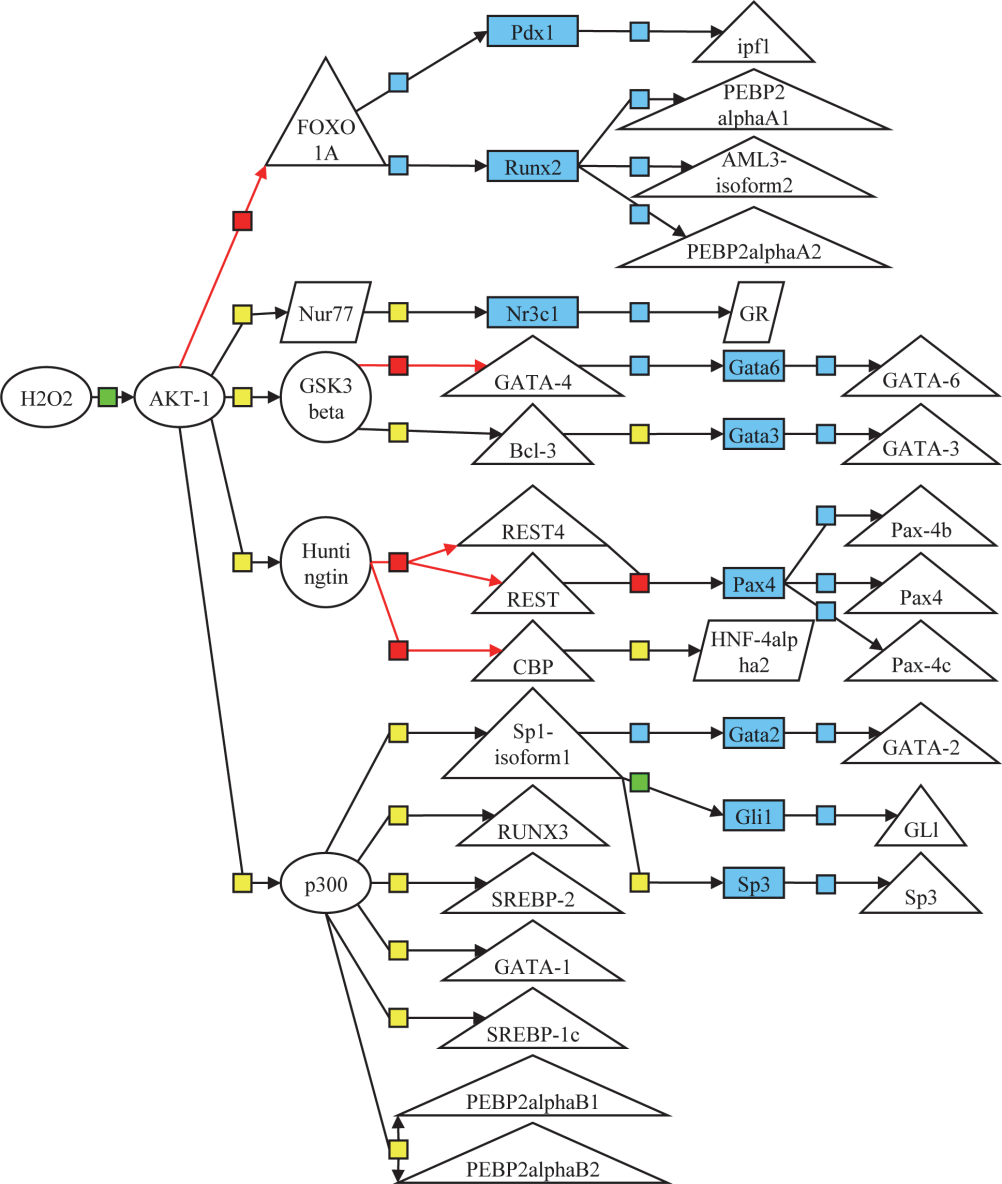
Gene cascade analysis in MAECs. The pathway map acquired from gene cascade analyses of quercetin-treated (50 μM) and untreated MAECs is shown. Black line + blue box represents enzymatic reaction or binding, red line + red box represents inhibition, black line + green box represents activation, and black line + yellow box represents other reactions. Diamond-shaped metabolites and proteins represent ligands, triangular proteins represent transcription factors, and blue rectangles represent genes.

To comprehensively detect the expression of genes associated with the mechanisms by which quercetin promotes salivary secretion, we conducted gene expression cascade analysis in MAECs, with the objective of evaluating the effects on peripheral circulation. TFBS analysis and key node analysis of the quercetin-treated and -untreated MAECs resulted in key nodes that were factors thought to cause changes in the expression of genes related to the binding sites. From several cascade networks derived from these key nodes, factors related to oxidative stress, such as Forkhead box protein O1A (FOXO1A), AKT-1 and H_2_O_2_, were detected with high frequency (Fig. 7). These findings indicate that quercetin regulates oxidative stress.

## Discussion

The aims of the present study were two-fold: to determine the effects of quercetin, a polyphenol, on salivary secretion and to elucidate its mechanism of action. Our investigation demonstrates that quercetin suppresses oxidative stress and proinflammatory cytokines and contributes to salivary secretion through the upregulation of AQP5 in gland cells.

Although we did not observe marked histological changes or clear reductions in the number of CD31+ cells in the SMGs of irradiated and quercetin-fed mice (data not shown), a previous report on radiation-induced impaired salivary secretion found that unlike our study, histology images showed inflammatory changes in the SMG, such as loss of acinar cells, vasodilation and angiogenesis at 4 weeks after irradiation [11]. Nonetheless, there have also been reports that did not show a clear and marked change [19, 20]. For example, Mishima et al. demonstrated that at 8 weeks after irradiation, the decrease in salivary secretion levels is not accompanied by obvious histological changes [21]. Therefore, the observations are not consistent, and it is unclear whether this is due to differences in the site or method of irradiation. In any case, it appears to be difficult to evaluate the glandular secretion capacity based on morphological/histological images.

It has been previously shown that inflammatory responses are involved in the impairment of salivary secretion by radiation [22, 23]. Our study also showed that TNF-α and IL-10 expression is markedly upregulated by irradiation, suggesting that salivary secretion was impaired through TNF-α-mediated inflammation. The anti-inflammatory cytokine IL-10 also increased with irradiation; however, it has previously been reported that the overexpression of TNF-α at inflammatory sites leads to increases in IL-10 to suppress TNF-α [24, 25]. Thus, IL-10 appears to increase in response to TNF-α expression.

*In vivo* experiments revealed that SMG AQP5 gene expression increased markedly with quercetin intake and that M3R gene expression showed an increasing trend. These results indicate that quercetin affects AQP5 and M3R molecules on glandular epithelial cells, thereby enhancing salivary secretion. Aquaporins are a class of membrane proteins that regulate the cell membrane’s permeability to water, and AQP5 is known to be localized in the salivary glands [26]. The neurotransmitter acetylcholine binds to M3R and activates G-proteins and phospholipase C, inducing the production of inositol triphosphate. This chain of events induces a cascade of movements in ions such as Ca^2+^, Cl^−^ and Na^+^, which in turn leads to the movement of water molecules through AQP5 [27, 28]. In AQP5 knockout mice, it has been shown that there is a ≥60% decrease in M3R agonist-stimulated salivary secretion [27], indicating that AQP5 plays a key role in this process. For this reason, investigations aimed at elucidating the detailed mechanisms of quercetin-induced AQP5 expression are currently underway.

Oxidative stress induced by radiation exposure is known to cause an impairment of various functional molecules [29, 30], and in our investigation, we showed that quercetin suppressed radiation-induced reductions in AQP5 expression levels, indicating that quercetin may have indirectly maintained AQP5 expression by eliminating radiation-induced oxidative stress.

The overproduction of reactive oxygen species (ROS) induced by radiation exposure is known not only to induce DNA damage but also to act on the cell membrane to induce lipid peroxidation, thereby impairing cellular function [31]. MDA, which is a degradation product of lipid peroxidation, is widely used as an indicator of lipid peroxidation [32]. In the irradiated mice in the present study, MDA decreased with quercetin intake, indicating that quercetin may have suppressed the radiation-induced generation of MDA. In a study on the effects of radiation exposure on SMGs, it was found that xerostomia can develop as an adverse event from the treatment of malignant tumors in the head and neck area [33], and clear salivary secretion impairment has also been shown in mouse salivary glands [4, 14]. Moreover, Sjögren’s syndrome patients are known to have high levels of oxidative stress markers such as 8-OHdG and HEL in their saliva [3], while oxidative stress generated from various environmental factors is also known to decrease salivary gland function [34]. Because it has also been indicated in mouse salivary glands that ROS is involved in radiation-induced reductions in salivary secretion [14], it is believed that regulating this oxidative stress may be effective in ameliorating the disease state. Tai et al. demonstrated in irradiated mice that salivary secretion is improved upon the administration of the antioxidant enzyme SOD [14]. In addition, it has been reported that MDA decreases in the SMGs from irradiated mice fed resveratrol, a polyphenol similar to quercetin, and that the radiation-induced reductions in salivary secretion levels in these mice were suppressed by resveratrol [11]. Combining these results with our findings, the data suggest that radiation-induced oxidative stress was eliminated by quercetin and that reductions in salivary secretion may have been suppressed as a result.

Furthermore, oxidative stress-related genes, such as FOXO1A and AKT-1, and H_2_O_2_ were detected upon cascade analysis of MAECs. FOXO is known to be involved in the control of cell death and aging caused by oxidative stress [35, 36], and phosphorylation by AKT is reported to be involved in suppressing this transcription factor [37]. In addition, H_2_O_2_ is a reactive oxygen species; therefore, quercetin may be involved in the regulation of oxidative damage.

In previous reports regarding the effects of quercetin on promoting salivary secretion in the present study, resveratrol, a polyphenol similarly to quercetin, did not show such effects [11], suggesting that quercetin possesses properties that directly stimulate salivary secretion. In the present in vitro experiments, quercetin treatment augmented calcium uptake, indicating that salivary secretion may have been promoted due to quercetin directly acting on molecules for secretion, such as AQP5 in the salivary gland. A previous study found that AQP5 gene expression is increased by M3R binding to some types of agonists [38], also suggesting that quercetin functions as an agonist of M3R.

[Ca^2+^]_i_ is intimately involved in the regulation of secretion capacity by muscarinic receptor stimulation in the acinar cells of the salivary gland [39]. Our findings showed that quercetin treatment augmented calcium uptake and did not upregulate M3R expression; therefore, molecules other than M3R may have triggered the increase in intracellular calcium concentration. It has been recently reported that acinar cells in the SMG with upregulated transient receptor potential canonical 1 (TRPC1), which is a receptor-activated Ca^2+^ channel (RACC), and stromal interaction molecule 1 (STIM1), both of which are expressed in the salivary gland, show augmented Ca^2+^ flow [40, 41]. It has also been reported in a calcium uptake study using 100 μM cch, similar to our experiment, that calcium concentration increases in salivary gland cells transfected with TRPC1 and STIM1 [40, 41]. These findings suggest that quercetin affected such channels in the present study, and we therefore plan to conduct a detailed analysis in the future.

It has been reported that improved blood flow causes increased salivary secretion [4]. Our results on irradiated-MAECs showed increased eNOS expression with quercetin treatment. As this increase in expression was not observed in non-irradiated cells, it is presumed that quercetin suppressed radiation-induced reductions in eNOS expression. A previous studies has indicated that radiation-induced reductions in eNOS expression are suppressed by statins that possess antioxidant abilities [30]. Combining this result with our findings from MDA and gene cascade analyses, the data suggest that quercetin indirectly restored eNOS expression by eliminating oxidative stress. For the functional analysis of quercetin’s effects on blood flow improvement in the SMG through its vasodilatory effects, we plan on conducting a detailed analysis in the future using equipment such as a laser Doppler blood flowmeter.

## Conclusion

In the present study, we showed that quercetin augments AQP5 expression as well as calcium uptake, thereby promoting salivary secretion. Furthermore, quercetin suppresses radiation-induced oxidative stress and inflammatory responses, thereby alleviating impaired salivary secretion. These results suggest that quercetin intake not only improves impaired salivary secretion caused by radiation exposure but may also be an effective method to maintain healthy salivary secretion.
